# Echocardiographic Guidance for Percutaneous Left Atrial Appendage Occlusion: A Systematic Review of Outcomes in High-Risk Populations Including Chronic Liver Disease and Prior Gastrointestinal Bleeding

**DOI:** 10.3390/diagnostics16050678

**Published:** 2026-02-26

**Authors:** Tina Becic, Ivana Jukić, Petra Šimac Prižmić, Ivona Matulić, Hana Đogaš, Mislav Radić, Josipa Radić, Jonatan Vuković, Damir Fabijanić

**Affiliations:** 1 Department of Cardiovascular Diseases, University Hospital of Split, 21000 Split, Croatia; tina.becic@gmail.com (T.B.);; 2 Division of Gastroenterology, Department of Internal Medicine, University Hospital of Split, 21000 Split, Croatia; 3 Faculty of Health Sciences, University of Split, 21000 Split, Croatia; 4 Division of Rheumatology, Allergology and Clinical Immunology, Department of Internal Medicine, University Hospital of Split, 21000 Split, Croatia; petra_simac@hotmail.com (P.Š.P.);; 5 Private Clinic Matulic, Osjecka Ulica 24a, 21000 Split, Croatia; ivonamatulic@yahoo.com; 6 Department of Neurology, University Hospital of Split, 21000 Split, Croatia; 7 Department of Internal Medicine, School of Medicine, University of Split, 21000 Split, Croatia; josiparadic1973@gmail.com; 8 Division of Nephrology, Dialysis and Arterial Hypertension, Department of Internal Medicine, University Hospital of Split, 21000 Split, Croatia; 9 Department of Clinical Propedeutics, School of Medicine, University of Split, 21000 Split, Croatia

**Keywords:** left atrial appendage occlusion, transesophageal echocardiography, intracardiac echocardiography, atrial fibrillation, chronic liver disease, gastrointestinal bleeding, structural heart interventions, peri-device leak

## Abstract

**Background**: Echocardiographic imaging has become central to planning and guiding percutaneous left atrial appendage occlusion (LAAO), particularly in patient populations in whom long-term anticoagulation is unsuitable. This systematic review synthesizes current evidence on transesophageal (TEE) and intracardiac echocardiography (ICE) guidance during LAAO, with special emphasis on outcomes in high-risk cohorts, including chronic liver disease (CLD) and prior gastrointestinal (GI) bleeding. **Methods**: Following PRISMA 2020 guidelines, four databases (PubMed, Scopus, Web of Science, and Cochrane CENTRAL) were searched up to 5 December 2025. Eligible studies included adult patients with atrial fibrillation (AF) undergoing percutaneous LAAO with intraprocedural echocardiographic guidance. Eight studies (*n* = 1739 patients) met the inclusion criteria. Data were synthesized qualitatively due to heterogeneity across devices, imaging protocols, and outcomes. **Results**: TEE was the predominant imaging modality (62.5%), providing high spatial resolution for transseptal puncture, device positioning, and peri-device leak (PDL) assessment. ICE-guided LAAO (25.0%) was associated with high procedural success and favorable safety profiles in selected observational cohorts, while reducing anesthesia requirements and fluoroscopy time. Across all studies, procedural success ranged from 93 to 100%, with low rates of major complications. Reported follow-up durations varied substantially across studies and were predominantly short- to mid-term, limiting assessment of long-term device-related outcomes. Evidence specific to patients with chronic liver disease and prior gastrointestinal bleeding was limited, with only two included studies directly evaluating these populations, while remaining insights were extrapolated from broader LAAO cohorts. In high-risk groups, LAAO remained feasible: cirrhotic patients demonstrated high implantation success with acceptable bleeding profiles, while patients with prior GI bleeding showed low recurrence after closure. **Conclusions**: Both TEE and ICE provide reliable intraprocedural imaging for LAAO, with ICE offering workflow and safety advantages in patients unsuitable for general anesthesia. The available evidence suggests that LAAO is a feasible and potentially safe therapeutic option in selected patients with CLD and prior GI bleeding, although direct data remain limited. Future studies should compare imaging modalities prospectively in high-risk cohorts and evaluate emerging 3D/4D ICE technologies.

## 1. Introduction

Echocardiography has undergone substantial technological and clinical transformation over the past decade, redefining its role in structural heart interventions and enabling increasingly precise, patient-tailored procedural guidance. This evolution is particularly evident in the imaging of the left atrial appendage (LAA), a key anatomical structure in the pathogenesis of thromboembolic events in atrial fibrillation (AF). Since early transesophageal echocardiography (TEE) studies demonstrated that the majority of thrombi in non-valvular AF originate within the LAA [1], advances in two- and three-dimensional imaging have substantially improved the understanding of LAA anatomy, functional dynamics, and pathophysiology [2,3].

As percutaneous left atrial appendage occlusion (LAAO) has become an established alternative to oral anticoagulation for stroke prevention—particularly in patients at high bleeding risk—echocardiography has become integral to all procedural phases. TEE has long served as the reference modality for pre-procedural assessment, intraprocedural guidance, and post-procedural evaluation, providing the spatial resolution and multiplanar visualization required for accurate device selection, transseptal puncture, positioning, and peri-device leak (PDL) assessment [4]. More recently, intracardiac echocardiography (ICE), initially used primarily in electrophysiological procedures, has gained acceptance as an alternative imaging modality for LAAO guidance. Observational data suggest that ICE-guided LAAO is feasible and associated with favorable procedural outcomes, while avoiding general anesthesia and potentially improving workflow efficiency [5,6].

The relevance of advanced echocardiographic guidance is particularly pronounced in clinically high-risk populations, for whom LAAO is often considered because long-term oral anticoagulation is ineffective or unsafe. Patients with chronic liver disease (CLD) represent a paradigmatic example, characterized by rebalanced hemostasis with concurrent bleeding and thrombotic risk, rendering conventional anticoagulation strategies challenging [7,8]. Similarly, patients with AF and prior gastrointestinal bleeding constitute a growing subgroup in whom recurrent bleeding frequently limits anticoagulant therapy. Notably, direct evidence evaluating echocardiographic guidance for LAAO specifically in patients with chronic liver disease or prior gastrointestinal bleeding remains limited, and current clinical insights in these populations are largely informed by extrapolation from general LAAO cohorts. In both populations, emerging data suggest that LAAO can be performed with acceptable procedural safety; however, direct evidence remains limited and is largely derived from broader, unselected LAAO cohorts [9,10].

Despite rapid advances in imaging technology—including three-dimensional ICE, multimodal fusion imaging, and artificial intelligence-assisted analysis—important gaps remain in understanding how echocardiographic guidance influences procedural outcomes across diverse patient profiles and anatomical substrates. Existing studies vary widely in imaging protocols, device selection, and operator experience, and no prior systematic review has specifically synthesized the evidence on TEE- and ICE-guided LAAO with attention to clinically vulnerable populations such as patients with CLD or prior gastrointestinal bleeding. In contrast to recent imaging-focused reviews that primarily address technical or procedural aspects of echocardiographic guidance for left atrial appendage occlusion, the present systematic review places imaging strategy within a patient-centered clinical framework, with particular emphasis on high-risk populations, including patients with chronic liver disease and those with prior gastrointestinal bleeding. In these populations, imaging modality selection may have disproportionate implications for procedural safety, anesthesia tolerance, and clinical outcomes.

Accordingly, the objective of this systematic review is to synthesize available evidence on echocardiographic guidance for percutaneous LAAO—including TEE, ICE, and emerging advanced modalities—with particular emphasis on procedural performance and safety in high-risk populations such as patients with chronic liver disease and prior gastrointestinal bleeding, while acknowledging that population-specific conclusions are informed by limited direct evidence and extrapolation from broader cohorts.

## 2. Materials and Methods

### 2.1. Study Design

This systematic review was conducted in accordance with the Preferred Reporting Items for Systematic Reviews and Meta-Analyses (PRISMA 2020) guidelines [11]. A completed PRISMA 2020 checklist is provided as Supplementary Materials. The review was designed to primarily evaluate contemporary echocardiographic guidance strategies for percutaneous left atrial appendage occlusion (LAAO) across clinical practice. High-risk populations, including patients with chronic liver disease and those with prior gastrointestinal bleeding, were examined as clinically relevant contextual subgroups, rather than as mandatory inclusion criteria, to explore the potential implications of imaging modality selection in vulnerable patients. This approach allowed for the synthesis of imaging performance and procedural outcomes while acknowledging that population-specific conclusions are informed by limited direct evidence. The review protocol was prospectively registered in PROSPERO (registration ID: CRD420251269086).

Given the heterogeneity of study designs, imaging protocols, and reported outcomes, as well as the limited number of studies directly evaluating high-risk populations, this review was not intended to perform a direct comparative effectiveness analysis between TEE and ICE. Accordingly, a qualitative narrative synthesis was undertaken to contextualize imaging performance and procedural outcomes, particularly within vulnerable patient subgroups.

### 2.2. Search Strategy

A comprehensive literature search was performed across four electronic databases: PubMed, Web of Science, Scopus, and Cochrane CENTRAL. The search covered all publications from database inception to the final search date, 5 December 2025. No language restrictions were applied. The search strategy used a combination of controlled vocabulary terms and free-text keywords related to LAAO, echocardiographic imaging modalities (TEE and ICE), and high-risk clinical subsets relevant to anticoagulation management. The complete search string applied to all databases was (“left atrial appendage” OR “left atrial appendage occlusion” OR “left atrial appendage closure” OR “LAA occlusion” OR “LAA closure” OR LAAO OR LAAC OR Watchman OR Amulet OR “Amplatzer Amulet” OR LAmbre) AND (echocardiograph OR “echo guidance” OR “intraprocedural imaging” OR “transesophageal echocardiography” OR “transoesophageal echocardiography” OR TEE OR “intracardiac echocardiography” OR ICE OR “3D ICE” OR “4D ICE” OR “fusion imaging” OR “image fusion”) AND ((“chronic liver disease” OR “liver cirrhosis” OR cirrhosis OR cirrhotic OR “hepatic failure” OR “portal hypertension” OR ACLD) OR ((“atrial fibrillation” OR AF OR “nonvalvular atrial fibrillation”) AND (“liver cirrhosis” OR cirrhosis OR “chronic liver disease” OR ACLD OR “hepatic failure”) AND (anticoagulant OR anticoagulation OR DOAC OR NOAC OR warfarin OR “vitamin K antagonist”))). The search strategy was intentionally broad with respect to echocardiographic imaging modalities and LAAO techniques. Terms related to chronic liver disease and gastrointestinal bleeding were included to ensure identification of studies involving high-risk populations; however, eligibility was not restricted to studies explicitly enrolling these subgroups. High-risk populations were analyzed as secondary, contextual subgroups within the overall synthesis.

Reference lists of all included studies and relevant reviews were also screened manually to ensure completeness. The search produced 573 records, which were systematically organized, evaluated, and screened to ensure adherence to PRISMA standards.

### 2.3. Study Selection

After removal of duplicates (*n* = 72), 501 records remained for title and abstract screening. Two reviewers (TB and PŠP) independently performed screening using predefined eligibility criteria, with discrepancies resolved by discussion.

A total of 111 full-text articles were evaluated. Of these, 103 studies were excluded for the following reasons:

- Population not meeting inclusion criteria (*n* = 34);
- Not a percutaneous LAAO intervention (*n* = 16);
- No relevant echocardiographic guidance (*n* = 9);
- Insufficient procedural details (*n* = 3);
- Duplicate full-text (*n* = 2);
- Case reports (*n* = 14);
- Case series (*n* = 10);
- Procedural/technical studies with a sample size < 5 (*n* = 15).

A total of 8 studies met all inclusion criteria and were included in the qualitative synthesis.

**Figure 1:**
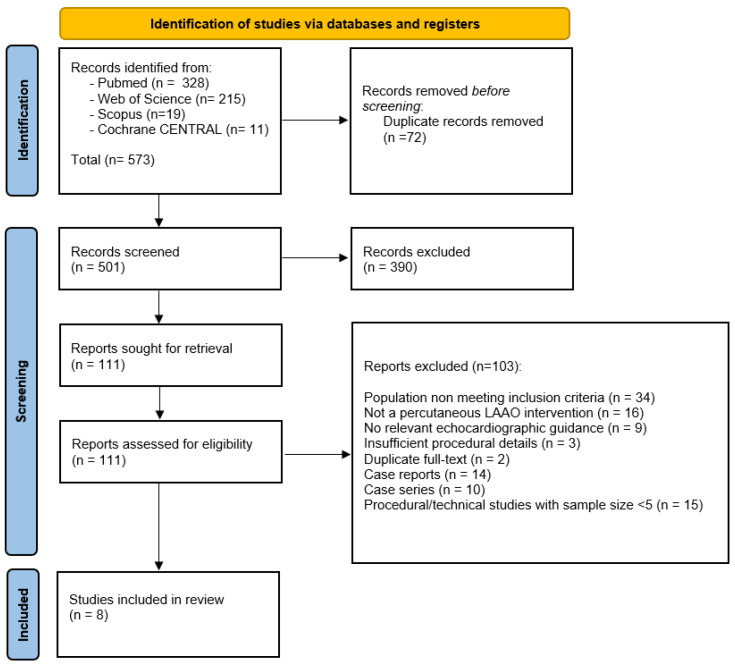
PRISMA 2020 flow diagram showing the selection process for included studies. Of the 573 records identified across the four databases, 72 duplicates were removed, leaving 501 records for screening. Following title and abstract review and full-text assessment, 8 studies fulfilled all eligibility criteria and were included in the qualitative synthesis.

A PRISMA flow diagram summarizing the selection process is provided in Figure 1.

### 2.4. Eligibility Criteria

Studies were considered eligible if they included adult patients with atrial fibrillation (paroxysmal, persistent, or permanent) undergoing percutaneous LAAO. Only studies that incorporated intraprocedural echocardiographic guidance—either TEE or ICE—and that reported procedural, imaging, or clinical outcomes were included. Eligible study designs comprised prospective or retrospective cohort studies, registries, early feasibility studies, and technical evaluations enrolling at least five participants. Studies including high-risk populations, such as patients with chronic liver disease or prior gastrointestinal bleeding, were also eligible but not mandatory for inclusion. Exclusion criteria encompassed surgical or thoracoscopic LAA closure procedures; case reports or small case series with fewer than five patients; studies lacking echocardiographic imaging during the procedure; non-human investigations; and non-peer-reviewed materials such as editorials, commentaries, and conference abstracts.

### 2.5. Data Extraction

Two reviewers (TB and PŠP) independently extracted data using a predefined, standardized form. The extracted variables included study design, geographic location, sample size, and baseline characteristics of the study population. Information on the type of intraprocedural echocardiographic guidance (TEE or ICE), device used (WATCHMAN, Amulet, PLAATO, Conformal Seal), and key procedural outcomes—such as procedural success, complications, and imaging-derived metrics including PDL—was collected. Follow-up duration and reported clinical outcomes were also recorded. Any uncertainties during data extraction were resolved by consensus between the two reviewers.

The extracted study characteristics are presented in Table 1.

### 2.6. Risk-of-Bias Assessment

Risk of bias in the included non-randomized studies was assessed using the ROBINS-I tool, evaluating seven domains: confounding, selection of participants, classification of interventions, deviations from intended interventions, missing data, measurement of outcomes, and selection of reported results. Most studies demonstrated a moderate-to-serious risk of bias, primarily related to residual confounding and limitations inherent to observational research. Interventions and outcome measurements were generally classified as low-risk.

Full domain-level assessments are provided in Table 2.

### 2.7. Data Synthesis

Because the included studies varied substantially in terms of devices used, imaging approaches, patient populations, and outcome definitions, the findings were integrated using a qualitative narrative synthesis. The analysis examined the relative performance of TEE and ICE guidance in procedural workflows, their impact on fluoroscopy requirements, and their role in detecting peri-device leaks and other imaging-derived outcomes. Particular attention was given to feasibility and safety profiles in high-risk subgroups, including patients with chronic liver disease and those with a history of gastrointestinal bleeding. Due to substantial heterogeneity across devices, imaging modalities, and outcome definitions, a meta-analysis was not feasible. The methodological approach described above enabled a comprehensive synthesis of the available evidence on echocardiographic guidance in percutaneous LAAO. The results of the eight studies that met all eligibility criteria are presented below.

## 3. Results

### 3.1. Study Characteristics

**Figure 2:**
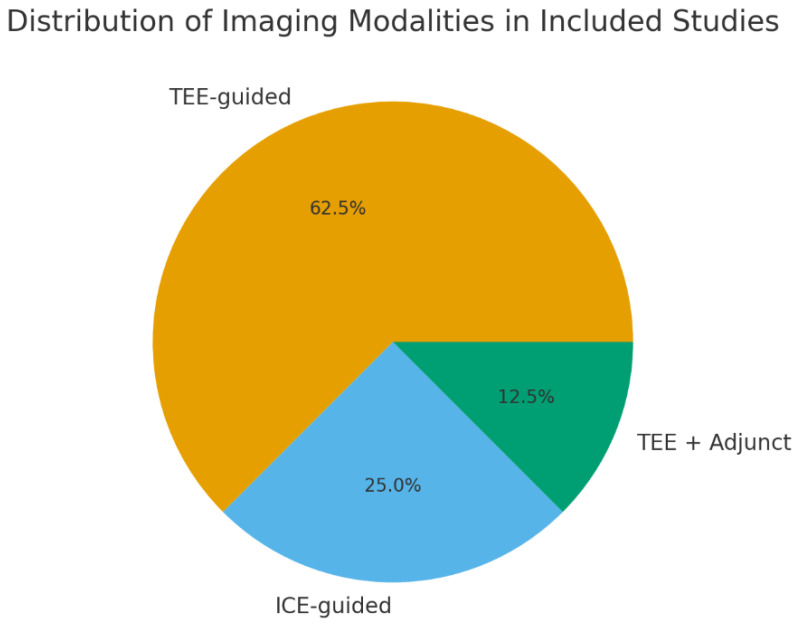
Distribution of imaging modalities used across the eight included studies. TEE was the predominant modality (62.5%), followed by ICE (25.0%) and combined TEE with adjunct imaging techniques (12.5%).

Eight studies published between 2004 and 2024 were included in the qualitative synthesis, encompassing a total of 1739 patients undergoing percutaneous LAAO. Study designs ranged from early feasibility investigations and technical evaluations to retrospective and prospective cohort analyses. Imaging modalities varied across studies, with five studies utilizing TEE as the primary intraprocedural modality, two focusing on ICE-guided LAAO, and one incorporating adjunct imaging techniques in the assessment of a novel conformal LAA seal device. The distribution of imaging modalities is presented in Figure 2. The WATCHMAN device was the most frequently used, followed by Amulet, PLAATO, and emerging conformable seal technologies. Detailed characteristics of the included studies are provided in Table 1.

### 3.2. Echocardiographic Guidance Modalities

#### 3.2.1. TEE-Guided LAAO

**Figure 3:**
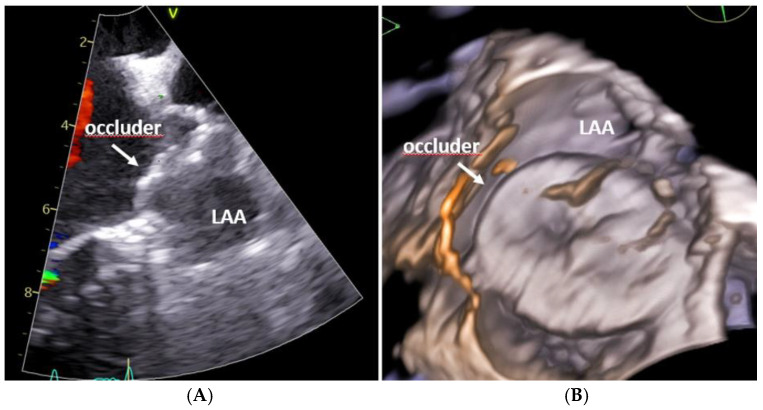
Representative echocardiographic imaging of left atrial appendage occlusion: (**A**) Two-dimensional transesophageal echocardiography showing the occlude positioned within the left atrial appendage (arrow). (**B**) Three-dimensional echocardiographic reconstruction illustrating device–appendage interaction and spatial orientation. LAA, left atrial appendage.

TEE remained the predominant imaging modality across the included studies. The TEE guidance provided reliable visualization of device positioning, peri-device leak (PDL) assessment, and procedural endpoints. Studies by Kefer et al., Haertel et al., and Kikuchi et al. consistently demonstrated high procedural success rates and low complication profiles with TEE-guided left atrial appendage occlusion (LAAO) [4,10,12]. Notably, Haertel et al. further reported that elevated galectin-3 levels were predictive of residual PDL, highlighting the utility of TEE in identifying subtle anatomical or tissue-related factors that may influence procedural efficacy and completeness of closure [12]. A representative example of intraprocedural echocardiographic visualization during TEE-guided left atrial appendage occlusion is shown in Figure 3.

#### 3.2.2. ICE-Guided LAAO

Two studies [5,6] evaluated ICE-guided LAAO. ICE enabled real-time intraprocedural imaging without the need for general anesthesia and was associated with high procedural success and favorable safety profiles in observational cohorts. Direct comparisons with TEE should be interpreted cautiously, given the non-randomized design of the available studies and potential confounding by operator experience, center volume, and device selection. In both studies, ICE guidance was associated with reduced fluoroscopy time and a streamlined procedural workflow. Additional feasibility experience with ICE-guided implantation has been summarized by Díaz et al. (2024), further illustrating the expanding integration of ICE into contemporary LAAO workflows [16].

### 3.3. Procedural Success and Complications

Procedural success rates were uniformly high across all included studies, ranging from 93% to 100%. Major adverse events, including pericardial effusion, device embolization, and clinically significant bleeding, were infrequent. Notably, the large cohort study by Mir et al. (*n* = 905) demonstrated that favorable procedural outcomes and acceptable safety profiles were maintained even among patients with chronic liver disease [9]. Across the studies, the incidence of peri-device leaks was generally low and was predominantly identified using high-quality intraprocedural imaging with transesophageal echocardiography (TEE) or intracardiac echocardiography (ICE). Reported procedural success rates should be interpreted in the context of potential selection and reporting bias inherent to observational studies, as well as limited and heterogeneous follow-up durations. These factors may overestimate procedural performance and limit the assessment of late device-related complications.

### 3.4. Imaging Performance and Peri-Device Leak Detection

TEE enabled comprehensive assessment of device apposition and residual flow, while ICE provided adequate visualization of the LAA ostium and device–wall interface. Studies evaluating PDL [6,12,17] highlighted that imaging quality directly influenced leak detection, particularly with newer, conformable seal devices. No study demonstrated a clear superiority of one modality over the other in preventing leaks; rather, imaging performance appeared device- and operator-dependent.

### 3.5. Outcomes in High-Risk Populations

Two included studies specifically investigated populations considered high-risk for anticoagulation-related complications.

- **Chronic liver disease [Mir et al.]**: Patients with cirrhosis undergoing LAAO experienced high procedural success with acceptable bleeding and mortality profiles, supporting LAAO as a viable alternative to oral anticoagulation in this group [9].
- **Prior gastrointestinal bleeding [Kikuchi et al.]**: Recurrent GI bleeding after LAAO was uncommon, and procedural safety was comparable to general LAAO populations [10].

These findings support the feasibility of LAAO—whether TEE- or ICE-guided—in patient groups traditionally considered challenging due to increased procedural risk.

### 3.6. Risk of Bias

Risk-of-bias assessment using ROBINS-I revealed that most studies were subject to moderate to serious risk of bias, primarily due to confounding and limitations inherent to observational study designs. However, classification of interventions, measurement of outcomes, and reporting completeness were generally assessed as low risk. Full domain-level assessments are summarized in Table 2.

### 3.7. Summary of Main Findings

Across eight included studies, TEE remained the most widely used imaging modality for LAAO guidance, while ICE-guided LAAO was associated with high procedural success and acceptable safety in selected cohorts, supporting its role as a viable alternative imaging strategy in appropriately selected patients. Procedural success was consistently high, peri-device leaks were generally infrequent, and major complications were rare. Importantly, the evidence supports the feasibility and safety of LAAO in high-risk groups, including patients with chronic liver disease and those with prior gastrointestinal bleeding.

## 4. Discussion

This systematic review integrates two converging domains: the safety, feasibility, and clinical effectiveness of left atrial appendage occlusion in high-risk populations—particularly patients with chronic liver disease and those with a history of gastrointestinal bleeding—and the evolving role of echocardiographic guidance, encompassing transesophageal echocardiography, intracardiac echocardiography, and advanced three- and four-dimensional imaging modalities, which increasingly influence procedural outcomes in contemporary structural heart interventions. While several recent publications have comprehensively examined echocardiographic imaging modalities for LAAO from a technical standpoint, they have largely focused on unselected or general patient populations. In contrast, our synthesis highlights how clinical vulnerability modifies the relevance and interpretation of imaging guidance, underscoring the need to align imaging modality selection with patient-specific risk profiles rather than relying solely on procedural preference or institutional routine. The synthesized evidence indicates that modern echocardiography has evolved beyond a passive visualization technique to become an active determinant of procedural success, especially in patient populations characterized by limited safety margins and complex anatomical substrates.

### 4.1. LAAO in CLD: Hemostatic Rebalancing and Clinical Implications

Patients with CLD represent one of the most challenging populations for stroke prevention in atrial fibrillation due to the pathophysiology of rebalanced hemostasis, where pro-bleeding and pro-thrombotic tendencies coexist in dynamic equilibrium [7,8,18,19]. Conventional coagulation tests frequently misrepresent bleeding risk in cirrhosis, complicating anticoagulation decisions [8,20,21]. Real-world studies further show heterogeneous outcomes with both DOACs and vitamin K antagonists [22,23,24,25,26,27,28], reinforcing the attractiveness of LAAO as an alternative stroke-prevention strategy in this cohort. The largest national cohort available [9] demonstrated high procedural success in cirrhotic patients undergoing LAAO, albeit with increased in-hospital mortality compared to non-cirrhotic individuals. Editorials and corroborating registry data [29,30] emphasize the importance of careful patient selection and multidisciplinary planning, rather than contraindicating LAAO outright. Collectively, these findings highlight LAAO as a clinically meaningful option for cirrhotic patients in whom anticoagulation is either unsafe or ineffective. However, despite the rising frequency of LAAO in CLD, there remains no study dedicated to evaluating how imaging modality selection influences outcomes specifically in this cohort—a significant gap given the unique anesthetic, anatomic, and hemodynamic challenges of advanced liver disease. An important methodological consideration in interpreting outcomes in patients with chronic liver disease relates to imaging granularity. In the largest available CLD cohort by Mir et al. [9], intraprocedural imaging was reported as “standard procedural imaging” without specification of the echocardiographic modality used. This lack of modality-level detail precludes imaging-specific interpretation of outcomes in this population and limits conclusions regarding the relative contribution of TEE or ICE to procedural success and safety. These findings underscore the need for future studies in CLD populations to report imaging strategies with greater precision.

### 4.2. LAAO in Patients with Prior Gastrointestinal Bleeding and Other High-Risk Populations

Among patients with prior gastrointestinal bleeding, the evidence shows consistently low recurrence rates following LAAO and a safety profile similar to the general LAAO population [10]. Additional high-risk groups—such as cancer patients [31,32], chronic kidney disease [33,34,35], and frail and elderly patients [36,37,38]—show similar procedural success despite elevated baseline risk. These parallel findings support a broader conceptual framework: when procedural imaging is optimized, LAAO remains feasible even in vulnerable populations, reinforcing the clinical relevance of advanced echocardiographic strategies.

### 4.3. TEE as the Historical and Contemporary Standard for LAAO Guidance

TEE has historically been the gold standard for LAAO planning, intraprocedural guidance, and follow-up due to its high spatial resolution and multiplanar visualization [1,2,3,4]. TEE allows for precise transseptal puncture localization, real-time device–tissue interaction assessment, and reliable PDL detection—features that continue to anchor its role in complex anatomies. TEE-based studies included in this review consistently demonstrated high procedural success, low complication rates, and reliable imaging endpoints [4,10,12,13,15]. The prognostic association between biomarkers such as galectin-3 and PDL [12] reinforces the ability of high-quality TEE to detect clinically relevant anatomic nuances. Nonetheless, reliance on general anesthesia represents a significant limitation, particularly in cirrhotic patients prone to hepatic encephalopathy, altered drug clearance, and hemodynamic instability [18,19,20]. These considerations have catalyzed expanded interest in ICE-guided LAAO. Despite its established role, TEE has important procedural limitations in patients with chronic liver disease and prior gastrointestinal bleeding. In cirrhotic patients, general anesthesia and deep sedation required for TEE may increase the risk of hepatic encephalopathy, hemodynamic instability, and altered drug metabolism, while coagulopathy and portal hypertension may further complicate transseptal access and periprocedural bleeding risk. In patients with a history of gastrointestinal bleeding, prolonged esophageal instrumentation and sedation may be poorly tolerated, particularly in frail individuals or those with recent hemorrhagic events. These considerations underscore the clinical relevance of alternative imaging strategies, such as ICE, in carefully selected high-risk patients.

### 4.4. ICE as a Transformative Imaging Strategy: Feasibility, Performance, and Clinical Impact

ICE offers several procedural advantages: elimination of general anesthesia, real-time catheter-based visualization from within the heart, reduced fluoroscopy exposure, and a streamlined workflow. Early comparative observational studies [5,6,16,17,39] suggest that ICE-guided LAAO is feasible and associated with high procedural success and favorable safety profiles, with potential gains in procedural efficiency.

More recent advances—including 3D ICE, 4D ICE, and fusion imaging technologies—have expanded ICE from a feasibility tool into a high-precision imaging modality. Novel work on 3D ICE for LAA sizing and intraprocedural assessment [40,41,42,43,44,45,46,47] demonstrates the capability to replicate, and in specific aspects enhance, TEE’s diagnostic performance. Furthermore, evidence suggests 3D/4D ICE may improve PDL detection and device–wall interface assessment, addressing historical limitations of 2D ICE. As these technologies mature, ICE is poised to become a first-line imaging modality, particularly in high-risk cohorts where avoidance of sedation is strongly advantageous. Importantly, the apparent similarity in procedural success between ICE- and TEE-guided LAAO reported in observational studies should not be interpreted as evidence of equivalence, as imaging modality selection is closely intertwined with operator expertise, institutional workflow, and device familiarity. Consequently, comparative effectiveness between imaging modalities cannot be definitively established based on the currently available evidence. The risk-of-bias assessment further informs the interpretation of these findings. Most included studies were subject to a moderate-to-serious risk of bias, primarily driven by residual confounding inherent to observational designs. Factors such as operator experience, center volume, institutional workflow, and device selection are closely intertwined with imaging modality choice and may substantially influence procedural outcomes. Consequently, associations observed between imaging modality and procedural success or safety should be interpreted as reflecting feasibility within experienced centers rather than intrinsic superiority of TEE or ICE.

### 4.5. Unmet Needs and Imaging Challenges Unique to Chronic Liver Disease

Despite the accumulating evidence for ICE, none of the available studies stratify imaging performance specifically in CLD patients. This represents a major unmet need because cirrhosis introduces procedural factors that place greater demand on imaging precision: altered interatrial septal compliance from volume overload, enlarged right atrium due to portal hypertension, hemodynamic instability under sedation, increased bleeding risk during transseptal puncture, and potential variability in LAA morphology due to chronic systemic changes. Guidelines and consensus documents [48,49,50,51,52] increasingly emphasize individualized imaging selection, yet no algorithm currently integrates hepatic disease severity, anesthesia tolerance, and intraprocedural imaging needs into a unified framework. Given the rising prevalence of LAAO in CLD and the signal for increased periprocedural morbidity in this group [9,29,30], future research must explicitly evaluate whether ICE can mitigate procedural risk by avoiding sedation and improving intraprocedural visualization in this fragile population.

### 4.6. Future Research Priorities

This review identifies several critical knowledge gaps that should inform future research on echocardiographic guidance for left atrial appendage occlusion. Most notably, there is a paucity of prospective studies directly comparing transesophageal echocardiography and intracardiac echocardiography in patients with chronic liver disease. Well-designed randomized trials or propensity-matched observational studies are required to determine whether the choice of imaging modality influences procedural safety, efficiency, and clinical outcomes in this particularly vulnerable population.

Further investigation is warranted to define the clinical utility of emerging three- and four-dimensional ICE technologies, especially in patients with complex left atrial anatomy, portal hypertension, or limited tolerance for general anesthesia. In parallel, greater emphasis should be placed on multimodality imaging strategies that combine preprocedural cardiac computed tomography with intraprocedural ICE and real-time fusion imaging, as these approaches may enhance device sizing and positioning while reducing the incidence of peri-device leaks.

Long-term outcome data in patients with cirrhosis undergoing LAAO remain limited. Future studies should address device-related thrombus formation, endothelialization processes, and late peri-device leaks, ideally within integrated hepatology–cardiology care models that account for disease severity and underlying hemodynamic alterations.

In summary, while LAAO appears feasible and generally safe in high-risk patient populations, substantial evidence gaps persist. As intracardiac echocardiography technologies continue to advance, the development of patient-tailored, evidence-based imaging strategies—particularly for individuals with chronic liver disease—will be essential to further optimize procedural outcomes and long-term clinical benefit.

Moreover, standardization of echocardiographic protocols and reporting criteria across imaging modalities will be essential to ensure reproducibility and comparability in future studies. Incorporating advanced quantitative imaging metrics and automated analysis tools may further refine risk stratification and procedural planning in high-risk patients. Importantly, multidisciplinary collaboration between interventional cardiologists, imaging specialists, and hepatologists should be prioritized to align procedural decision-making with systemic disease burden. Collectively, these efforts will be critical for translating technological advances in echocardiography into meaningful improvements in patient-centered outcomes.

## 5. Clinical Implications and Future Perspectives

The findings of this review support a personalized, patient-centered approach to imaging modality selection during left atrial appendage occlusion (LAAO). Transesophageal echocardiography remains indispensable in cases involving complex appendage anatomy, detailed peri-device leak assessment, and the deployment of newer or investigational devices that require high-resolution structural imaging. Intracardiac echocardiography offers particular advantages in patients who are poor candidates for general anesthesia, in centers seeking to enhance procedural efficiency, and in clinical settings where operator expertise with ICE is well established. Looking ahead, the integration of preprocedural cardiac computed tomography, advanced three-dimensional ICE imaging, artificial intelligence-assisted segmentation, and real-time fusion imaging technologies is likely to define the next phase of procedural optimization in LAAO.

Despite these advances, the current evidence base remains constrained by the predominance of observational study designs, heterogeneity in imaging protocols, and limited long-term follow-up. The absence of standardized imaging acquisition and reporting criteria further complicates cross-study comparisons and outcome interpretation. Future research should therefore prioritize randomized comparisons of imaging modalities, the development of device-specific imaging algorithms, and standardized definitions of procedural success and residual PDL. In addition, the incorporation of imaging-derived quantitative metrics may enhance procedural planning and risk stratification. Long-term, multicenter studies focusing on anatomically and clinically complex patient populations will be essential to determine the durability and clinical relevance of these imaging-guided strategies.

## 6. Limitations

This systematic review has several limitations. First, the included studies were predominantly observational and varied substantially in design, sample size, and reporting standards, which limits the ability to draw causal inferences. Importantly, evidence specific to patients with chronic liver disease and prior gastrointestinal bleeding remains limited, with only two of the included studies directly evaluating these populations. Consequently, conclusions pertaining to these high-risk subgroups should be interpreted as supportive rather than definitive and are largely informed by extrapolation from broader, unselected LAAO cohorts. Second, significant heterogeneity across imaging modalities, devices, patient populations, and outcome definitions precluded the performance of a quantitative meta-analysis. Third, echocardiographic protocols were not standardized across studies, introducing variability in peri-device leak assessment and other imaging-dependent outcomes. Fourth, most studies reported short- to mid-term follow-up, limiting interpretation of long-term device performance and clinical outcomes. Finally, publication bias cannot be excluded, particularly given the small number of studies evaluating ICE-guided procedures and high-risk subgroups.

## 7. Conclusions

Transesophageal echocardiography remains the cornerstone imaging modality for guiding left atrial appendage occlusion, supported by extensive clinical experience and its ability to provide high-resolution, comprehensive visualization of key procedural endpoints. Its established role in device sizing, positioning, and peri-device leak assessment continues to make TEE indispensable, particularly in anatomically complex cases and during the deployment of novel or investigational devices. Intracardiac echocardiography has emerged as an increasingly credible and practical alternative, especially for patients who are poor candidates for general anesthesia, and its clinical utility is expected to expand further with the maturation of three-dimensional and four-dimensional ICE technologies.

Across both imaging modalities, LAAO is associated with consistently high procedural success rates and low complication profiles, including in high-risk populations such as patients with chronic liver disease or a history of gastrointestinal bleeding. These findings underscore the feasibility of LAAO even in clinically fragile cohorts when appropriate imaging guidance is employed. Importantly, the choice of imaging modality appears to influence procedural workflow efficiency, resource utilization, and patient tolerability, reinforcing the need for individualized imaging strategies rather than a uniform approach.

Ongoing advances in echocardiographic technology, device design, and multimodality imaging integration are likely to further refine procedural precision and safety. The incorporation of preprocedural cardiac computed tomography, refined three-dimensional ICE imaging, and real-time fusion imaging may enhance anatomical understanding, optimize device selection, and reduce residual PDL. In parallel, artificial intelligence-driven image analysis and quantitative assessment tools hold promise for improving procedural planning and standardizing outcome evaluation.

Nevertheless, current evidence remains limited by the predominance of observational data, heterogeneity in imaging protocols, and a relative scarcity of long-term outcome studies in high-risk patient populations. Addressing these limitations through prospective, methodologically robust investigations will be essential to establishing evidence-based imaging algorithms. These findings support a shift from modality-centered to patient-centered imaging strategies, particularly in populations with limited procedural safety margins, such as those with chronic liver disease or prior gastrointestinal bleeding. Ultimately, continued innovation coupled with multidisciplinary collaboration will be key to expanding the applicability of LAAO and improving long-term outcomes in anatomically and clinically complex patients.

## Figures and Tables

**Table 1:** Summary of the key characteristics of the eight studies included in the qualitative synthesis.

Study (Year)	Study Design	Population	Imaging Modality	LAAO Device/Technique	Sample Size (n)	Key Outcomes
Mir et al., 2023 [9]	Retrospective national cohort	Patients with cirrhosis and AF undergoing LAAO	Standard procedural imaging	WATCHMAN/Amulet (NR)	905	Procedural success, bleeding, stroke, mortality
Kikuchi et al., 2024 [10]	Retrospective cohort	Patients with AF and prior GI bleeding undergoing LAAO	TEE	WATCHMAN	115	Predictors of GI bleeding recurrence; procedural safety
Kefer et al., 2018 [4]	Multicenter registry	AF patients undergoing percutaneous LAAO	TEE	WATCHMAN, Amulet	457	Procedural success, complications, follow-up outcomes
Haertel et al., 2023 [12]	Prospective observational	AF patients undergoing LAAO	TEE	WATCHMAN	102	Galectin-3 as predictor of peri-device leak
Block, 2004 [13]	Early clinical series	AF patients unable to take warfarin	TEE	PLAATO device	15	Feasibility, procedural success, early safety
Grazina et al., 2023 [6]	Retrospective cohort	AF patients undergoing LAAO with ICE guidance	ICE	WATCHMAN/Amulet	123	Procedural efficiency, safety, fluoroscopy reduction
Frazzetto et al., 2024 [14]	Technical study	Patients undergoing ICE-guided LAAO	ICE	WATCHMAN	12	Feasibility, safety, simplified ICE workflow
Sommer et al., 2021 [15]	First-in-human clinical evaluation	AF patients undergoing novel conformal LAAO	TEE + adjunct imaging	Conformal LAA Seal	10	Feasibility, device seal performance

Abbreviations: AF, atrial fibrillation; GI, gastrointestinal; ICE, intracardiac echocardiography; LAA, left atrial appendage; LAAO, left atrial appendage occlusion; NR, not reported; TEE, transesophageal echocardiography.

**Table 2:** Summary of the risk-of-bias assessment for the included studies using the ROBINS-I tool.

Study	Bias Due to Confounding	Bias in Selection of Participants	Bias in Classification of Interventions	Bias Due to Deviations from Intended Interventions	Bias Due to Missing Data	Bias in Measurement of Outcomes	Bias in Selection of Reported Results	Overall ROB
Mir et al., 2023 [9]	Serious	Moderate	Low	Low	Low	Low	Moderate	Serious
Kikuchi et al., 2024 [10]	Serious	Moderate	Low	Low	Low	Low	Moderate	Serious
Kefer et al., 2018 [4]	Moderate	Moderate	Low	Low	Moderate	Low	Moderate	Moderate
Haertel et al., 2023 [12]	Serious	Moderate	Low	Low	Moderate	Low	Moderate	Serious
Block, 2004 [13]	Serious	Serious	Low	Low	Moderate	Low	Serious	Serious
Grazina et al., 2023 [6]	Moderate	Moderate	Low	Low	Low	Low	Moderate	Moderate
Frazzetto et al., 2024 [14]	Serious	Moderate	Low	Low	Low	Low	Moderate	Serious
Sommer et al., 2021 [15]	Moderate	Moderate	Low	Low	Low	Low	Moderate	Moderate

Abbreviations: ROB, risk of bias; ROBINS-I, Risk of Bias in Non-randomized Studies of Interventions.

## Data Availability

No new data were created or analyzed in this study. Data sharing is not applicable to this article.

## Supplementary Materials

The following supporting information can be downloaded at: https://www.mdpi.com/article/10.3390/diagnostics16050678/s1, Table S1: PRISMA 2020 Checklist [11].

## Abbreviations

The following abbreviations are used in this manuscript:

AF	Atrial Fibrillation
AI	Artificial Intelligence
CLD	Chronic Liver Disease
CT	Computed Tomography
DOAC	Direct Oral Anticoagulant
GI	Gastrointestinal
ICE	Intracardiac Echocardiography
LAA	Left Atrial Appendage
LAAC	Left Atrial Appendage Closure
LAAO	Left Atrial Appendage Occlusion
PDL	Peri-Device Leak
PRISMA	Preferred Reporting Items for Systematic Reviews and Meta-Analyses
ROBINS-I	Risk of Bias in Non-randomized Studies of Interventions
TEE	Transesophageal Echocardiography
3D	Three-Dimensional
4D	Four-Dimensional
