# Supplementary material for: Echocardiographic Guidance for Percutaneous Left Atrial Appendage Occlusion: A Systematic Review of Outcomes in High-Risk Populations Including Chronic Liver Disease and Prior Gastrointestinal Bleeding
Source: Diagnostics (Basel). 2026 Feb 26;16(5):678. doi: 10.3390/diagnostics16050678 (PMC12984305; doi:10.3390/diagnostics16050678)
Supplement: Supplementary file 1 [file diagnostics-16-00678-s001.zip › diagnostics-4091911-supplementary.pdf]

PRISMA 2020 Checklist

Section and Topic	Item #	Checklist item	Location where item is reported
**TITLE**			
Title	1	**The report is explicitly identified as a systematic review in the title.** The title states: *"Echocardiographic Guidance for Percutaneous Left Atrial Appendage Occlusion: A Systematic Review of Outcomes in High-Risk Populations Including Chronic Liver Disease and Prior Gastrointestinal Bleeding."*	Title
**ABSTRACT**			
Abstract	2	**The abstract follows PRISMA 2020 for Abstracts.** It includes a structured summary with Background, Methods (databases searched, eligibility criteria, PRISMA adherence), Results (number of included studies and patients, key findings), and Conclusions (implications for imaging modality selection and high-risk populations).	Abstract
**INTRODUCTION**			
Rationale	3	**The rationale for the review is clearly described in the context of existing knowledge.** The introduction explains the evolving role of echocardiography in structural heart interventions, the established role of TEE, emerging use of ICE, and the lack of synthesized evidence comparing imaging strategies during LAAO—particularly in high-risk populations such as patients with chronic liver disease and prior gastrointestinal bleeding. The review addresses an important knowledge gap regarding optimal intraprocedural imaging in vulnerable patient groups.	Introduction
Objectives	4	**The objectives of the review are explicitly stated.** The primary objective is to synthesize available evidence on echocardiographic guidance (TEE, ICE, and advanced modalities) during percutaneous LAAO. A secondary objective is to evaluate procedural and clinical outcomes in high-risk populations, specifically patients with chronic liver disease and those with a history of gastrointestinal bleeding.	Introduction (final paragraph)
**METHODS**			
Eligibility criteria	5	**Eligibility criteria were predefined.** Included studies enrolled adult patients with atrial fibrillation undergoing percutaneous left atrial appendage occlusion with intraprocedural echocardiographic guidance (TEE or ICE) and reported procedural, imaging, or clinical outcomes. Eligible designs included prospective or retrospective cohort studies, registries, early feasibility studies, and technical evaluations enrolling ≥5 patients. Studies involving high-risk populations (e.g., chronic liver disease or prior gastrointestinal bleeding) were eligible but not mandatory. Exclusion criteria included surgical or thoracoscopic LAA closure, case reports or series <5 patients, studies without echocardiographic guidance, non-human studies, editorials, conference abstracts, and non–peer-reviewed publications.	Methods – Eligibility Criteria
Information sources	6	**Four electronic databases were searched:** PubMed, Web of Science, Scopus, and Cochrane CENTRAL. Searches covered database inception to 5 December 2025. No language restrictions were applied. Reference lists of included studies and relevant reviews were manually screened to identify additional eligible studies.	Methods – Search Strategy
Search strategy	7	**A comprehensive search strategy combining controlled vocabulary and free-text terms was used.** Search terms addressed LAAO, echocardiographic guidance (TEE, ICE, 3D/4D ICE, fusion imaging), and high-risk populations relevant to anticoagulation management. The complete search string is fully reported in the Methods section and was applied consistently across all databases, without filters or limits.	Methods – Search Strategy
Selection process	8	**Study selection was performed independently by two reviewers.** After duplicate removal, titles and abstracts were screened independently by two reviewers using predefined eligibility criteria. Full texts of potentially eligible studies were reviewed independently. Discrepancies were resolved by discussion and consensus. No automation tools were used during the screening process.	Methods – Study Selection
Data collection process	9	**Data extraction was conducted independently by two reviewers using a standardized form.** Extracted data included study design, population characteristics, imaging modality, device type, procedural outcomes, imaging-derived outcomes, and follow-up data. Any uncertainties or discrepancies were resolved by consensus. No direct contact with study investigators or automation tools was required.	Methods – Data Extraction
Data items	10a	**Primary outcomes included procedural success, safety outcomes (major complications), and imaging-derived endpoints such as peri-device leak.** Secondary outcomes included fluoroscopy time, anesthesia requirements, and clinical outcomes during follow-up (bleeding, stroke, mortality). All reported outcomes compatible with these domains were collected as reported in individual studies.	Methods – Data Extraction
	10b	**Additional variables collected included study design, sample size, geographic location, patient characteristics, imaging modality**	Methods –

PRISMA

PRISMA 2020 Checklist

Section and Topic	Item #	Checklist item	Location where item is reported
		**(TEE or ICE), device type, and follow-up duration.** Funding sources were recorded when reported. No assumptions were made for missing or unclear data; such data were reported as not available.	Data Extraction
Study risk of bias assessment	11	**Risk of bias was assessed using the ROBINS-I tool for non-randomized studies.** Two reviewers independently assessed seven bias domains (confounding, selection, classification of interventions, deviations, missing data, outcome measurement, selective reporting). Disagreements were resolved by consensus. No automation tools were used.	Methods – Risk of Bias Assessment
Effect measures	12	**Due to substantial heterogeneity, no quantitative effect measures were synthesized.** Outcomes were summarized descriptively using proportions, ranges, and narrative comparison across studies. No pooled effect estimates were calculated.	Methods – Data Synthesis
Synthesis methods	13a	**Studies were included in the qualitative synthesis if they met all predefined eligibility criteria.** Studies were grouped descriptively according to imaging modality (TEE vs. ICE), device type, and inclusion of high-risk populations.	Methods – Data Synthesis
	13b	**No statistical transformations or imputation methods were applied.** Data were extracted and presented as reported in the original studies. Missing summary statistics were not estimated.	Methods – Data Synthesis
	13c	**Results were presented using narrative synthesis, structured tables summarizing study characteristics, and figures illustrating study selection and imaging modality distribution.** A PRISMA 2020 flow diagram was used to depict the selection process.	Methods – Results Presentation
	13d	**Results were synthesized using qualitative narrative synthesis.** Meta-analysis was not performed due to substantial heterogeneity in study design, imaging modalities, devices, populations, and outcome definitions. Studies were compared descriptively according to imaging modality (TEE vs. ICE), procedural performance, and outcomes in high-risk populations. No statistical software was used for quantitative synthesis.	Methods – Data Synthesis
	13e	**Formal statistical exploration of heterogeneity was not performed.** Clinical and methodological heterogeneity was explored qualitatively by comparing imaging modalities, study designs, device types, and inclusion of high-risk populations (e.g., chronic liver disease, prior gastrointestinal bleeding).	Methods – Data Synthesis
	13f	**No sensitivity analyses were conducted.** The limited number of studies and the qualitative nature of the synthesis precluded sensitivity or robustness analyses.	Methods – Data Synthesis
Reporting bias assessment	14	**Risk of bias due to missing results or reporting bias was not formally assessed.** Funnel plot analysis and statistical tests for publication bias were not appropriate given the small number of included studies and absence of meta-analysis.	Methods – Limitations
Certainty assessment	15	**Certainty of evidence was not formally assessed using tools such as GRADE.** This was due to the observational nature of included studies, heterogeneity of outcomes, and absence of pooled quantitative estimates.	Methods – Limitations
**RESULTS**			
Study selection	16a	**The study selection process is described in detail.** A total of 573 records were identified across four databases. After removal of 72 duplicates, 501 records underwent title and abstract screening. Following full-text review of 111 articles, 8 studies met all eligibility criteria and were included in the qualitative synthesis. The selection process is illustrated using a PRISMA 2020 flow diagram.	Results – Study Selection; Figure 1
	16b	**Reasons for exclusion of full-text articles are explicitly reported.** Exclusions included ineligible populations, lack of echocardiographic guidance, non-percutaneous interventions, insufficient procedural detail, case reports or small case series, and duplicate publications.	Results – Study Selection
Study characteristics	17	**Characteristics of all included studies are presented.** Reported variables include study design, population, imaging modality, device type, sample size, and key outcomes. These data are summarized in a structured table.	Results – Study Characteristics; Table 1
Risk of bias in studies	18	**Risk of bias assessments for each included study are presented using the ROBINS-I tool.** Most studies demonstrated moderate to serious risk of bias, primarily due to confounding and observational design limitations. Domain-level and overall judgments are summarized in a dedicated table.	Results – Risk of Bias; Table 2
Results of	19	**Results of individual studies are reported descriptively.** Summary statistics such as procedural success rates, complication rates, and	Results –

PRISMA

PRISMA 2020 Checklist

Section and Topic	Item #	Checklist item	Location where item is reported
individual studies		imaging findings (e.g., peri-device leak) are presented for each study. Effect estimates with precision were not calculated due to heterogeneity and qualitative synthesis.	Sections 3.1–3.5; Table 1
Results of syntheses	20a	**For each synthesis, characteristics and risk of bias of contributing studies are summarized.** Findings are interpreted in the context of study design, imaging modality, and patient risk profile, with acknowledgment of moderate to serious risk of bias in most studies.	Results – Summary of Main Findings
	20b	**No statistical synthesis was performed.** Therefore, no pooled effect estimates, confidence intervals, or measures of statistical heterogeneity are reported.	Results – Data Synthesis
	20c	**Investigations of possible causes of heterogeneity were conducted qualitatively.** Variability in results was explored by comparing study design, imaging modality (TEE vs. ICE), device type, operator experience, and inclusion of high-risk populations (e.g., chronic liver disease, prior gastrointestinal bleeding).	Results – Sections 3.2–3.5
	20d	**No sensitivity analyses were performed.** The limited number of studies and absence of quantitative synthesis precluded formal sensitivity analyses.	Results – Data Synthesis
Reporting biases	21	**Risk of bias due to missing results was not formally assessed.** Assessment of publication or reporting bias was not appropriate given the small number of included studies and the lack of meta-analysis.	Results – Risk of Bias; Limitations
Certainty of evidence	22	**Certainty (confidence) in the body of evidence was not formally assessed using GRADE or similar frameworks.** This decision reflects the observational nature of included studies, heterogeneity of outcomes, and qualitative synthesis approach.	Results – Limitations
**DISCUSSION**			
Discussion	23a	**The results are interpreted in the context of existing evidence.** The discussion integrates findings with prior literature on echocardiographic guidance for LAAO, highlighting the established role of TEE, emerging evidence supporting ICE, and the relevance of imaging selection in high-risk populations such as patients with chronic liver disease and prior gastrointestinal bleeding.	Discussion – Sections 4.1–4.4
	23b	**Limitations of the included evidence are discussed.** These include predominantly observational study designs, moderate to serious risk of bias, heterogeneity in imaging protocols and devices, small sample sizes for ICE studies, and limited long-term follow-up.	Discussion – Limitations
	23c	**Limitations of the review process are acknowledged.** These include exclusion of non–peer-reviewed literature, absence of quantitative synthesis, potential publication bias, and lack of standardized echocardiographic outcome definitions across studies.	Discussion – Limitations
	23d	**Implications for clinical practice and future research are discussed.** The findings support individualized imaging selection for LAAO and highlight the need for prospective comparative studies of TEE versus ICE, particularly in patients with chronic liver disease and other high-risk populations, as well as evaluation of emerging 3D/4D ICE technologies.	Discussion – Future Research Priorities
**OTHER INFORMATION**			
Registration and protocol	24a	**The review protocol was prospectively registered in PROSPERO.** The registration number is reported in the Methods section.	Methods – Study Design
	24b	The review protocol is accessible via the PROSPERO registry.	Methods – Study Design
	24c	No amendments were made to the protocol after registration.	Methods – Study Design
Support	25	**The review received no external financial support.** The funders had no role in study design, data collection, analysis, interpretation, or manuscript preparation.	Funding

PRISMA 2020 Checklist

Section and Topic	Item #	Checklist item	Location where item is reported
Competing interests	26	**The authors declare no competing interests.**	Conflicts of Interest
Availability of data, code and other materials	27	**No new data or analytic code were generated.** All data used in the review are derived from published studies and are available within the article and referenced literature.	Data Availability Statement

From: Page MJ, McKenzie JE, Bossuyt PM, Boutron I, Hoffmann TC, Mulrow CD, et al. The PRISMA 2020 statement: an updated guideline for reporting systematic reviews. BMJ 2021;372:n71. doi: 10.1136/bmj.n71.
This work is licensed under CC BY 4.0. To view a copy of this license, visit https://creativecommons.org/licenses/by/4.0/
